# Problem-Based Learning Research in Anesthesia Teaching: Current Status and Future Perspective

**DOI:** 10.1155/2014/263948

**Published:** 2014-05-29

**Authors:** G. Chilkoti, M. Mohta, R. Wadhwa, A. K. Saxena

**Affiliations:** ^1^Department of Anesthesiology and Critical Care, University College of Medical Sciences and Guru Teg Bahadur Hospital, Shahdara, Delhi, India; ^2^A-1404, Jaipuria Sunrise Greens, Ahinsa Khand, Indirapuram, Ghaziabad, Uttar Pradesh 201014, India

## Abstract

The teaching curriculum in anesthesia involves traditional teaching methods like topic-based didactic lectures, seminars, and journal clubs; intraoperative apprenticeship; and problem-based learning (PBL) and simulation. The advantages of incorporating PBL in anesthesia teaching include development of skills like clinical reasoning, critical thinking, and self-directed learning; in addition it also helps in developing a broader perspective of case scenarios. The present paper discusses the characteristics, key elements, and goals of PBL; various PBL methods available; lacunae in the existing knowledge of PBL research; its current status and future perspectives in anesthesia teaching.

## 1. Introduction

The postgraduate (PG) medical education and training in anesthesia have undergone advanced transformation in the last decade due to growing interest in anesthesia and pain management specialty, easily accessible internet services, and availability of various learning and skill acquisition courses. The teaching curriculum in anesthesia involves traditional teaching methods like topic-based didactic lectures, seminars, and journal clubs; intraoperative apprenticeship; and problem-based learning (PBL) and simulation. The traditional lecture-based approach is applied universally; however, it restricts the development of power of creativity, critical thinking, and reasoning skills as the learner plays a passive role in this approach [1]. Although simulation is considered a powerful generic tool for teaching and dealing with human performance issues (e.g., training, research), it is associated with many limitations like exorbitant cost, the need of infrastructure, and trained faculty [2]. PBL thus stands desirable as it is comparatively easier to implement and is readily accepted by the students [1]. Problem-based learning is a student-centered pedagogy in which students in small groups learn about a subject through the experience of problem solving. It is also defined as “active learning stimulated by and focused around a clinical or scientific problem” [3]. The key point is that learning commences as a problem that the learner seeks to solve [4, 5]. Problem-based learning is considered complex and heterogeneous as it constitutes a wide variety of educational methods as shown in Table 1 [6]. In classical or inquiry-based PBL, students are given a planned, contextualized patient problem along with the resources for the self-directed learning. Following this, the group formulates objectives and the students are allowed free enquiry in tutor led group [7]. Inquiry-based PBL has been studied to a limited extent in anesthesia and is not widely practiced in anesthesia PG teaching. Case studies or case-based discussion is also a type of PBL, which is most widely and routinely practiced in anesthesia PG teaching. In this method, a complete organized case is given to the students for study prior to class discussions, which is facilitated by a tutor and is a combination of student and teacher directed learning. The objective of this paper is to present PBL philosophy and taxonomy, to report on the PBL research in anesthesia, and to recommend new directions for further PBL research in anesthesia teaching. To our knowledge, no previous detailed reviews discussing the status of PBL research in anesthesia teaching have been published.

## 2. Literature Search

A search from the National Library of Medicine PubMed database was conducted up to February 2014 using the following key words: “problem based learning,” “anesthesia,” and “case based discussions.” PBL has been adopted in educational programs in various disciplines, but very few articles related to use of PBL in anesthesia teaching were retrieved. These included nine clinical studies and three reviews. Out of these nine studies, four and five were performed in undergraduate and postgraduate anesthesia teaching, respectively. Amongst the four articles in undergraduate teaching, three assessed the student satisfaction with PBL in comparison to traditional lecture-based method [1, 8, 9], and one assessed the educational objectives accomplished with PBL [10]. The relevant clinical studies in postgraduate teaching compared PBL with traditional lecture-based method only in specific topics in anesthesiology like ethical aspects [11], preanesthetic assessment [12], intensive care [13], and continued medical education on air embolism [14]. The fifth clinical study was a recently published, retrospective observational study evaluating the effect of implementation of PBL discussion format amongst PG students [15]. Out of the 3 reviews, one discussed the practical aspects of PBL implementation in intensive care unit (ICU) [16] and the second one, briefly, described the PBL and its key areas [17]. The third review documented the pedagogy of learning with case study methods in anesthesia teaching [18]. The reference lists of all the relevant articles have been scrutinized and included in this review paper.

## 3. Overview of PBL

PBL is an established teaching method in medical education. In the early 1960s, PBL was first developed and implemented at the McMaster Medical University in Canada by Barrow and Tamblyn, which was soon followed in Europe and Australia [19, 20]. The American Society of Anesthesiologists (ASA) has also included PBL discussions (PBLD) in its continuous medical education course since 1991 [21]. The key areas of PBL include small group discussions; learner-centered, active, or self-directed learning; learning objectives formulation; and facilitation and supplementation of inadequacies of small group discussions [21].

An acronym spelt “PROBLEMS” identifying the eight key features of PBL was one of the results of the symposium on PBL held at the Centre for Medical Education, the University of Dundee [22]. Small group, facilitation, and evaluation, which are the other important key elements for determining the success of PBL, were not included in the acronym. So herein, we have modified this acronym to “PROBLEMSS-EF” to include small group, evaluation, and facilitation in the same acronym. Thus the acronym spelt “PROBLEMSS-EF” identifies the eleven key features of PBL.

*Problems*. The key units for learning in PBL are problems.

*Resources*. Provision of adequate resources (e.g., instruction, peers, library, and Internet) allows self-directed learning.

*Objectives*. Learning objectives are planned by teachers and may have trainee input.

*Behavior*. Progressively evolving behavior with increasing knowledge is gained through PBL.

*Learning*. Learning is active, learner-centered, and self-directed.

*Examples*. Through the use of examples, high-order cognitive skills are facilitated.

*Motivation*. PBL design should stimulate interest in topic and motivate learning.

*Self-Directed Learning and Self-Assessment*. Trainees are given the tools or resources to undertake self-directed learning.

*Small Group*. To achieve the desired learning outcome, small group discussions have been made an integral component of PBL discussions. In order to achieve maximal development of communication skills and knowledge, it is proposed to have a group size of 5–10 members [23, 24].

*Evaluation Methods*. The assessment drives learning. There has been a lot of debate on the spectrum of assessment methods used for various PBL research works. Various methods used are multiple-choice questions, multiple-essay questions, and triple-jump exercise test. Multiple-choice questions are the most commonly used assessment method in PBL research. Multiple-essay questions are also an established method [25] but have been found to be misused or overused in various PBL programs [26]. Another widely used evaluative measure in PBL is triple-jump exercise test [27]. It consists of 3 steps: a patient problem-based structured oral examination, a time limited study assignment in relation to problem, and finally a repeat oral examination to assess the quality of self-directed learning during the study period.

*Facilitation*. The facilitation skill and knowledge of the facilitator are important factors influencing the success of PBL scenario. PBL is learner-centered and thus the role of a teacher is that of a facilitator/moderator. The facilitator plays a key role as he/she not only monitors but also directs the PBL process [28]. A facilitator must restrict the limit to which he/she provides solution and yet provides the structure to obtain the maximum benefit. The role of facilitator is also to guard the group dynamic process. The best facilitator is the one who is content area expert and has been trained in facilitation and management of group dynamics [17, 29]. Group dynamics refers to the interactions between people in a group setting. A facilitator must be an expert in utilizing various techniques to manage the group dynamics. Different situations may require different techniques of managing group dynamics like equalizing participation, listing, stacking, pacing, checking the process, silence, taking a break, call for consensus, summarizing, censoring, expulsion, and so forth [29]. Since the role of teacher is of a facilitator than of a knowledge imparter, the scarcity of teaching faculty skilled in facilitation is a major limitation. There is increasing concern on the need of developing various programs for faculty training in facilitation.

## 4. PBL Taxonomy

The PBL taxonomy was originally proposed by Harden and Davis [30]. They argued that “PBL is considered as genus with many species and subspecies.” There is no strict application of PBL in medical education, but almost all disciplines include at least some component of PBL. The popular term PBL does not refer to a specific educational method. Inquiry-based is usually considered as the classical PBL method; however, case-based discussion, modified case-based method, case-based lecture, and lecture-based case have also been described as various types of PBL. Hybrid PBL incorporates PBL in addition to the lecture-based traditional teaching, for example, case-based lecture and lecture-based case [31].

Barrows in 1986 introduced four educational objectives that are possibly accomplished with various PBL methods [32]. It is important to know about these educational objectives as different PBL methods can have wide variation in accomplishment of these educational objectives resulting in variable learning outcome.

## 5. Barrow's Educational Objectives (see [32])

Educational objectives addressed by the various PBL programs are as follows.

*(1) Structuring of Knowledge for Use in Clinical Context (SCC)*. In PBL, learning must be integrated and structured along with the reasoning to solve patient problem in context of future task.

*(2) Development of Effective Clinical Reasoning Process (CRP)*. Clinical reasoning is an important skill for health professionals to achieve high level of expertise. This skill needs to be developed through repeated practice and feedback so that the problem solving skills and knowledge work together in clinical setting.

*(3) Development of Effective Self-Directed Learning Skills (SDL)*. This skill allows the students to identify personal learning needs and to locate and use the appropriate information resources. This is particularly important for the anesthetist and intensivist, as they often work alone and away from their peer group.

*(4) Increased Motivation for Learning (MOT)*. Motivation facilitates extraction and understanding of information from various resources, thus enhancing the learning and clinical performance. So, an educational method must provide enough motivation for learning.

Table 1 gives details about the PBL taxonomy and Barrow's rating of meeting the educational objectives. The inquiry-based PBL and closed loop PBL are given almost the maximum scoring for all the four objectives and thus, these are also termed as “upper-case PBL” methods. On the other hand, “lower-case PBl” includes an indefinite range of methods that give “problems” a central place [31]. In the anesthesia PG teaching, the most commonly used PBL design is case-based discussion [18]. Since the case material in this method is already organized, the educational objectives like structuring of knowledge for clinical use, self-directed learning, and clinical reasoning process are limited, whereas motivation for learning is nearly comparable to that of inquiry-based PBL.

## 6. PBL Research in Medical Education

Various authors have found PBL to be provocative with better learning results [33–36]. It has been observed that graduates of PBL curricula demonstrate superior clinical competencies than the traditional curricula [37]. Albanese and Mitchell, in their meta-analysis, concluded that PBL is more nurturing and enjoyable and the PBL graduates perform better on clinical evaluation [38]. Vernon and Blake conducted a meta-analysis on PBL research in medical education from 1970 till 1992. They examined PBL research works comparing PBL and traditional teaching method. They observed no difference in test of factual knowledge and clinical knowledge; however, PBL was found to be superior with respect to student's program evaluation [39].

After enough appraisal, Colliver [40], for the first time, criticized the PBL theory and highlighted the major problems in PBL research, that is, difference in effect sizes between PBL and traditional methods in various PBL meta-analyses and the educational theories and related research supporting PBL. Norman and Schmidt responded to Colliver's criticism and argued that “the problem lies with the programme evaluators, not the theoreticians” [41]. They emphasized that PBL has multiple components that interact synergistically and the evaluation method used must consider all the components along with their interactions. They strongly recommended that PBL research needs to be conducted in controlled, experimental conditions with evaluations to be done in real clinical setting with maximum efforts to involve all PBL variables.

## 7. PBL Research in Anesthesia

The implementation of PBL is highly desirable in the anesthesia teaching curriculum. According to Barrow's rating of meeting the educational objectives, inquiry-based PBL and closed loop PBL are considered the best, but these methods have been studied to a very limited extent in anesthesia. Till now, very few anesthesia departments worldwide have incorporated these “upper-case PBL” methods like inquiry-based and closed loop in their teaching curriculum.

In undergraduate anesthesia teaching, Chang et al. compared student satisfaction between the lecture-based traditional teaching and PBL and concluded that implementation of PBL for teaching in anesthesia showed satisfactory results; however, the assessment tools and content of PBL required further modification [1]. Grzeskowiak et al. [8] concluded that PBL is a better method of teaching basic life support (BLS) and advanced cardiac life support (ACLS) to undergraduate medical students than the classical lecture-based method. The assessment method used was multiple-choice question and clinical skill testing before and after the session. Carrero et al. compared two methods for teaching BLS algorithm to undergraduate medical students, that is, multimedia presentation and case-based method. The assessment methods used were scenario-based quiz and error-pinpointing video. The authors concluded that both teaching methods equally improved the level of cognitive skills among medical students. The limitation of both these studies using PBL for BLS teaching was small sample size.

Moret et al. [10] assessed the accomplishment of educational objectives with PBL in undergraduate anesthesia teaching. Educational objectives were defined and incorporated in 12 PBL cases. At the end of the sessions, the students underwent a voluntary and anonymous test to analyze the understanding and assimilation of knowledge. This study found PBL to be valid for meeting educational objectives; however, it recommended further studies to demonstrate the effect of PBL on academic results and assimilation of knowledge as long-term effect.

In PG teaching, only few studies have compared lecture-based approach and PBL discussions (“inquiry-based” PBL) for particular topics in anesthesia like preanesthetic checkup [11], ethical reasoning skills [12], and intensive care [13]. Carrero et al. used PBL for teaching the topic “preanesthetic assessment” and compared its effectiveness with the traditional lecture-based method by using an objective knowledge assessment tool before and after teaching [11]. Various aspects of assessment like recognizing, reasoning, memorizing, and selection were considered and scored separately in the assessment method. They concluded that implementation of PBL is a suitable teaching method for teaching preanesthetic assessment. de Oliveira Filho and Schonhorst [13] described the implementation of PBL in one-year introductory intensive care course to anesthesiology. The study was conducted for a period of two years. During the first year, the students were provided with lectures, demonstrations, and PBL, whereas in the subsequent year, only PBL was used for training students. On evaluation, no difference was observed between the courses and it was concluded that PBL may effectively address basic topics in anesthesiology during intensive care training. However, the limitation of this study was the lack of control group. Very recently, Sakai and colleagues, in a retrospective observational study, compared the education outcome amongst anesthesia PG students, before and after the implementation of PBL discussion format, and found it to be effective [15].

Case-based discussion is the most commonly practiced but the most understudied PBL method [42]. Very recently, Miclescue, in a review, discussed the PG anesthesia teaching curriculum in their medical university and concluded that case-based discussion is a popular, reliable, and satisfactory learning procedure despite its practical limitation regarding the selection of appropriate cases [18]. However, Grzeskowiak et al. could not demonstrate any advantage of case-based method over the traditional lecture method when used for teaching BLS algorithm to undergraduate students [8] as well as in continuing medical education on air embolism [14]. The effectiveness of case-based discussion and lecture-based method has been reported to be similar in terms of improving participants' immediate acquisition of knowledge, capacity of applying knowledge to solve problems, information management, clinical reasoning, or linking basic and clinical knowledge.

There may be a variation in case-based discussion setups of various institutions in terms of their adherence to the aforementioned key aspects of PBL. This is definitely a future potential area for performance measurement and improvement. In view of the limited research, further studies are needed to evaluate the role of case-based discussion in comparison to the traditional lecture-based method in anesthesia. Simultaneously, a broader and organized implementation of case-based discussion incorporating the key elements of PBL needs emphasis in anesthesia PG teaching curriculum.

In all PBL research work in anesthesia, there are significant variations in the designs of PBL and methods of assessment used. Some studies even lack the control group. As far as variation in the PBL design is concerned, there have been no claims for effectiveness of one particular model of PBL over another [43]. PBL is multidimensional and not a single entity; rather it is composed of many variables (aforementioned key areas of PBL) and their interactions may affect the results of employing a particular type of PBL. Most of the studies using inquiry-based PBL method have variations in terms of the sample size, objective formulation, assessment tool used, and so forth. A small sample size is the problem with most of the studies done in PG students and is practically difficult to solve. The use of pre- and postteaching session evaluation has been found to be a useful tool as an objective evidence of comparing the effectiveness of different educational methods [10]. Assessment methods in all these studies have measured knowledge primarily and have failed to measure attitude and communicative and clinical skills which are equally important aspects of clinical competence. Therefore, it is difficult to comment if PBL has really enhanced the participant's clinical competence.

## 8. Feasibility and Cost

Cost and feasibility are the important factors in determining the type of PBL method to be employed. Inquiry-based PBL and the closed loop PBL have the greatest education potential but require complex problem simulation for teaching and evaluation. These two PBL methods are difficult and require great effort from the administrative or curriculum designer in terms of the need to formulate objectives, problems selection, scheduling of time, and the development and maintenance of resources. These factors may be attributable to the limited research involving these methods in anesthesia teaching.

“Lower-case PBL” methods like lecture-based PBL methods are least expensive and most feasible as a single tutor can address many students simultaneously; however, their educational potential is lower.

## 9. Conclusions and Future Perspectives

An organized application of PBL in anesthesia teaching may help to accomplish the educational objectives. “Upper-case PBL” methods like inquiry-based PBL and closed loop PBL which address the educational objectives to the maximum degree need to be incorporated in anesthesia PG curriculum. An appropriate methodology for these methods needs to be developed and tested in larger number of students using standardized assessment tools in different topics in anesthesia.

There is a need for further research into the effects of different types of PBL programs on different levels of learners (undergraduates, postgraduates). In other words, we must determine which level of students benefit maximum with which PBL method. For example, inquiry-based PBL may provide better results in 1st year PG students, whereas case-based discussions may prove to be better in final year PG students.

Facilitation and management of group dynamics are the keys to success of PBL programs. Effective measures should be taken to train or enable the tutors to acquire facilitation and group dynamic management skills. Various techniques for group dynamics management need to be reinforced in order to help the group function effectively in PBL. Much research is needed to assess the effects of group dynamics on learning.

The main limitations of PBL research are its multidimensional nature and lack of reliable and valid measure for assessing the effect of PBL. Thus, there is need to develop high quality, systematic research programs with standardized evaluative measures assessing all its components and their interactions. In addition, it is also recommended to have long-term assessment of the effects of incorporating PBL on clinical performance in actual clinical practice in anesthesia.

## Figures and Tables

**Table 1:** Taxonomy of problem-based learning^#^.

Educational method	Description of method	Barrow's rating of meeting the educational objectives [32]			
		SCC	CRP	SDL	MO
Lecture-based case	(i) Information is presented as lectures first and then the cases are used to emphasize significant points	1	1	0	1
	(ii) Teacher directed learning				

Case-based lecture	(i) Cases are presented first for study prior to class lecture followed by the lecture covering the relevant area	2	2	0	2
	(ii) Teacher directed learning				

Case-based discussions	(i) A complete case is given to the student for study prior to class discussion, which is facilitated by a tutor	3	3	3	4
	(ii) Teacher directed and student directed				

Modified case based	(i) It features sequential management problems Students are given some information and asked to decide the action plan; following the conclusion, they are given more information	4	3	3	5

Problem or inquiry based	(i) Students are presented with a new patient problem and allowed free inquiry in tutor led group	4	4	4	5
	(ii) Teacher and student directed				

Closed loop or reiterative	(i) An extension of “inquiry-based PBL” method, in which after the initial problem solving, the students are asked to return to the original problem for reevaluation of their problem solving activities	5	5	5	5
	(ii) Both teacher and student directed				

Barrow's score 1 to 5 represents the likelihood (1: least and 5: most) that the educational method will meet the educational objectives. SCC: structured clinical context, CRP: clinical reasoning process, SLD: self- directed learning, and MOT: motivation for learning [32].

^#^Modified from Cisneros et al. [43].
